# Development and use of a master health facility list: Haiti's experience during the 2010 earthquake response

**DOI:** 10.9745/GHSP-D-14-00029

**Published:** 2014-08-05

**Authors:** Alyson Rose-Wood, Nathan Heard, Roody Thermidor, Jessica Chan, Fanor Joseph, Gerald Lerebours, Antonio Zugaldia, Kimberly Konkel, Michael Edwards, Bill Lang, Carmen-Rosa Torres

**Affiliations:** a U.S. Department of Health and Human Services, Office of Global Affairs, Washington, DC., USA; b U.S. Department of State, Humanitarian Information Unit, Washington, DC., USA; c Ministère de la Santé Publique et de la Population, Unité de Planification et d'Evaluation, Port-au-Prince., Haiti; d Pan American Health Organization, Emergency Operations Center, Washington, DC., USA; e MEASURE Evaluation, Chapel Hill, NC., USA; f U.S. Department of Health and Human Services, Center for Faith-Based and Neighborhood Partnerships, Washington, DC., USA; g Shoreland, Inc., Arlington, VA., USA

## Abstract

Master health facility lists (MHFLs) are gaining attention as a standards-based means to uniquely identify health facilities and to link facility-level data. The ability to reliably communicate information about specific health facilities can support an array of health system functions, such as routine reporting and emergency response operations. MHFLs support the alignment of donor-supported health information systems with county-owned systems. Recent World Health Organization draft guidance promotes the utility of MHFLs and outlines a process for list development and governance. Although the potential benefits of MHFLs are numerous and may seem obvious, there are few documented cases of MHFL construction and use. The international response to the 2010 Haiti earthquake provides an example of how governments, nongovernmental organizations, and others can collaborate within a framework of standards to build a more complete and accurate list of health facilities. Prior to the earthquake, the Haitian Ministry of Health (Ministère de la Santé Publique et de la Population [MSPP]) maintained a list of public-sector health facilities but lacked information on privately managed facilities. Following the earthquake, the MSPP worked with a multinational group to expand the completeness and accuracy of the list of health facilities, including information on post-quake operational status. This list later proved useful in the response to the cholera epidemic and is now incorporated into the MSPP's routine health information system. Haiti's experience demonstrates the utility of MHFL formation and use in crisis as well as in the routine function of the health information system.

## Abstract (precis)

Collaboration between the Haitian government and NGOs after the 2010 earthquake contributed to a more accurate and complete master health facility list, which helped coordinate emergency response operations as well as strengthen the routine health information system. Open data and social networks facilitated the collection and sharing of health facility information and in maintenance of the list over time.

## INTRODUCTION

Accurate and regularly updated master health facility lists (MHFLs) are essential for effective planning, coordination, and delivery of health services, particularly in low- and middle- income countries with extensive external donor presence. MHFLs are also important during disasters because the lack of accurate, usable information is a major obstacle to effective disaster response.^1^^,^^2^ According to the World Health Organization (WHO), an MHFL is a complete list of health facilities in a country (both public and private) with a set of attributes to uniquely identify each facility,^3^ and it includes basic information about the facility's services and capacities.

Lack of accurate information is a major obstacle to effective disaster response.

With the release in 2012 of draft WHO guidance on the creation and governance of MHFLs, countries may be considering devoting resources to develop such lists.^3^ However, there are few documented experiences on the construction or use of MHFLs.

The earthquake in Haiti on January 12, 2010, underscored the need for countries to have an MHFL. At the time of the earthquake, the Haitian Ministry of Health (Ministère de la Santé Publique et de la Population [MSPP]) had a list of public-sector health facilities. However, the MSPP list contained little information about privately managed health facilities—a major part of the health delivery system in Haiti—and it lacked critical attributes needed to uniquely identify facilities.

During the earthquake response, the MSPP worked with the Haiti Health Facilities Work Group (Work Group), composed of a multinational group of organizations and governments, to develop a functioning MHFL, which proved useful during not only the earthquake response but also subsequent events such as the cholera outbreak.

This article reviews the development and use of the MHFL and provides a model for other countries interested in developing similar lists, which are increasingly needed to align donor-supported information systems with national health information systems.

## THE NEED FOR A COMPLETE HEALTH FACILITY LIST IN HAITI

The epicenter of the 2010 Haiti earthquake was close to the most densely populated areas of Haiti, including the capital, Port-au-Prince. Approximately 250,000 buildings collapsed, including many hospitals.^4^ In the confusion that followed, there was uncertainty about the extent of damage to health facilities^5^ as well as a lack of information about the temporary clinics that were rapidly being set up. First responders were uncertain whether they were referring to the same health facility when communicating about the type, status, and capacity of facilities.

Master health facility lists help emergency response personnel know they are communicating about the same facility.

The MSPP is responsible for the health of the population and for the delivery of health-related services.^6^ At the time of the earthquake, the public health care system included more than 500 health institutions (approximately 30% of the country's health facilities), ranging from community health clinics providing basic primary services to university hospitals. In addition, there were more than 250 nongovernmental organizations (NGOs) providing a substantial proportion of the primary health services.^4^^,^^7^

Prior to the earthquake, multiple, incomplete, and conflicting health facility information systems existed in Haiti; no entity served as a repository for an up-to-date, comprehensive master list. For example, the Haiti Health Information System (Système d'Information Sanitaire d'Haïti [HSIS]) functioned as a health management information system but was incomplete. Similarly, the Electronic Monitoring, Evaluation and Surveillance Interface for HIV-infected patients (MESI) collected data from public, and some private, health facilities in Haiti but primarily from sites receiving support from the U.S. President's Emergency Plan for AIDS Relief (PEPFAR).

## DEVELOPMENT AND USE OF HAITI'S MASTER HEALTH FACILITY LIST

In the immediate aftermath of the earthquake, the U.S. Department of Health and Human Services (HHS) coordinated the formation of the Work Group in support of the MSPP. The first meeting of the Work Group took place via teleconference 5 days after the earthquake (on January 17, 2010) with representatives from U.S. federal agencies; academia; international, local, and Haitian diaspora NGOs; multilateral organizations; foundations; and businesses (Table 1). All Work Group activities were coordinated through conference calls and a shared web space.^8^ Staff of the MSPP's Planning and Evaluation Unit (Unité de Planification et d'Evaluation [UPE]) were responsible for the HSIS; they participated in the Work Group and had final determination regarding edits to the MHFL.

**Table 1.** Organizational Participation in the Haiti Health Facilities Work Group by Category (N = 56)

**Haitian Government**	**NGOs and Private Consultants**
Ministère de la Santé Publique et de la Population	Arkemie
**U.S. Government**	Association of Haitian Physicians Abroad, Florida Chapter
Department of Defense (DOD)	Baertracks
Department of Education	Christian Connections for International Health
Department of Health and Human Services	Christian Medical and Dental Associations
Department of Homeland Security	Citizen Command Center Database Team, Citizen Action Team
Department of State	Communibuild Technologies
Peace Corps	CrisisCommons
United States Southern Command (DOD)	DirectRelief
U.S. Agency for International Development	Evotech, Inc.
**Multilateral Organizations**	FortiusOne, Inc.
Geo-Operations Unit, United Nations	Global Health Action
International Organization for Migration (IOM)^a^	Haitian Mental Health Network
Office for the Coordination of Humanitarian Affairs, United Nations	Haiti Village Health
World Health Organization/Pan American Health Organization	Humanitarian Medical Aid Direct Relief
United Nations Development Programme	ICF International
World Health Organization headquarters	IMA World Health
**Academia**	InSteDD
Bloomberg School of Public Health, Johns Hopkins University	InterAction
Center for Geographic Analysis, Harvard University	Logistics for Health
Emory University	MEASURE Evaluation
The George Washington University	Medical Mission Exchange
Institute for Global Leadership, Tufts University	OpenStreetMap
Lincoln Laboratory, Massachusetts Institute of Technology	Project Medishare for Haiti
Mailman School of Public Health, Columbia University	ReliefWeb
**Foundations**	Sahana Software Foundation
Clinton Foundation	Shoreland, Inc.
Google Foundation	Synergist Technology Group, Inc.
	Thermopylae Sciences + Technology
	Ushahidi
	World Cares Center
	World Concern

a Although IOM is not part of the UN system, it works very closely with the UN specialized agencies and is part of UN Country Teams around the world.

Broad representation in the Haiti Health Facilities Work Group facilitated coordination while minimizing duplication of efforts.

Between January 2010 and August 2011, UPE staff collaborated with the Work Group to develop a single, comprehensive list of all public, private, and mixed (public and private) health facilities. The list was standardized, validated, and up-to-date to guide and coordinate the health response to the earthquake. Senior MSPP leadership provided input and direction during several meetings in 2010.

The MHFL's initial purpose was to address the urgent need for a common list of health facilities in Haiti to ensure all emergency response personnel knew they were communicating about the same facility. The Work Group adapted guidelines on a minimum set of data elements necessary to uniquely identify a health facility, known as a signature domain,^9^ to inform the core set of information contained in the MHFL (Box). These elements included:

- A unique identifying code for each facility
- The facility name, address, and type
- The entity that manages/owns the facility
- Each facility's geographic coordinates

**BOX. Recommended Data Elements for Master Health Facility Lists**

**Signature Domain** (set of data elements that do not change significantly over time)

- Unique identifier
- Facility name
- Facility type
- Ownership/managing authority
- Location/address
- Administrative units
- Geographic coordinates
- Operational status
- Year data collected

**Service Domain** (set of data elements that provide some basic information on a facility's services and capacities)

- Core basic services offered
- Number of core medical personnel
- Number of inpatient and maternity beds available

Adapted from the World Health Organization.^3^

A codebook for the signature domain fields was created building on codes created by the Institut Haïtien de Statistique et d'Informatique (Supplementary Appendix).

The first iteration of the MHFL that contained only the signature domain fields was created on January 29, 2010, by blending the MSPP's existing HSIS health facility list with partial lists, volunteered geographic information, and local knowledge on the post-earthquake status of health facilities in order to produce a more comprehensive list (Table 2). To improve functionality, the Work Group included standardized names of each facility in English, French, and Haitian Creole. The Work Group verified information by soliciting feedback on a publicly posted version of the MHFL coupled with direct outreach to health facilities by phone or in-person when possible.

**Table 2.** Evolution of the Haiti Master Health Facility List

**Version**	**Date**	**Host**	**Edits/Additions**	**Comments**
Pre-earthquake	Before 2010	The HSIS list was available online through the HSIS website.	Last updated in 2009.	No entity served as a repository for an MHFL. The HSIS became the basis for the MHFL, but it was incomplete; it did not cover the non-public sector and had variable reporting from the 750 public health facilities in it.
1	January 29, 2010	PAHO	Information on health facilities from the HSIS was merged with other lists creating a total of +/- 1,260 records. The 2009 HSIS health facility list included the following fields: rank (a number assigned to the facility according to when it was created in the commune); name of the department, district, and commune where the facility is located; name of the facility; category (e.g., dispensary, hospital); and type (public, private, or mixed).	Information sources included: HSIS, MESI, USAID, PAHO, PEPFAR, UNOCHA, the Sahana Foundation, MINUSTAH, and Ushahidi.
2	February 9, 2010	PAHO	7 new health facilities were added.	
3	February 12, 2010	PAHO	New fields were added for damage and operational status information; 39 new health facilities were added (including field hospitals); and 41 duplicate records were removed.	
4	February 16, 2010	PAHO	63 new health facilities were added; 19 duplicate records were removed; and metadata was updated.	
5	February 26, 2010	PAHO	HealthC_IDs (unique identification codes) were added to facilities that previously lacked one.	All HealthC_IDs from version 4 remained unchanged, but changes were made to the algorithm used to generate new unique identifiers in the metadata (Supplementary Appendix).
6	March 11, 2010	PAHO	Region, commune, and department IDs in the MHFL were matched to the MSPP_2010 list; official facility names were added; inaccurate values for the various codes used by the MSPP were corrected; geocodes of numerous MSPP sites were corrected; and about 20 duplicate records were removed. This version included all 2010 MSPP health facilities.	New information received post-earthquake from MEASURE Evaluation on behalf of the Haitian MSPP was incorporated into the new MHFL.
7	March 18, 2010	PAHO. Stewardship transferred to Shoreland, Inc., in April 2010, and version 7 was republished on Shoreland's Travax system.^17^	50 duplicates were removed and more than 80 new health facilities were added. CATEGORIE, TYPE, and SANTE_ID fields were updated with the latest information from the MSPP.	When the list was republished on the Travax site,^17^ a field for cholera treatment centers was added.
Liste des Institutions Sanitaires	Summer 2011	MSPP	Health facilities and field hospitals can now be uniquely identified. Data are updated and validated through self-reporting from facilities and data collection efforts by the MSPP and partners. Some duplicates and data quality issues remain. The list does not include information on mobile clinics (those that are still operational).	The MSPP incorporated the MHFL for a key input to its routine health information system. The MHFL forms the basis of the Liste des Institutions Sanitaires^19^ and is integrated into the MSPP's service delivery and infrastructure database.^20^^,^^21^ It is used on an ongoing basis to measure health service coverage.

Abbreviations: HSIS, Haiti Health Information System (Système d'Information Sanitaire d'Haïti); MESI, Electronic Monitoring, Evaluation and Surveillance Interface for HIV-infected patients; MHFL, master health facility list; MINUSTAH, United Nations Stabilization Mission in Haiti; MSPP, Haitian Ministry of Health (Ministère de la Santé Publique et de la Population); PAHO, Pan American Health Organization; PEPFAR, U.S. President's Emergency Plan for AIDS Relief; UNOCHA, United Nations/Office for the Coordination of Humanitarian Affairs; USAID, U.S. Agency for International Development.

In keeping with United Nations recommendations on the coordination of information during humanitarian emergencies, the Pan American Health Organization's Emergency Operations Center (PAHO EOC) took a lead role in managing the MHFL.^10^^,^^11^ The PAHO EOC published the first iteration of the MHFL and codebook to a public Google Site.^12^ Posting the list to an open website increased the likelihood that the MHFL and its codes would be used and that those involved in the response would provide feedback to note missing facilities and to correct errors. Contributors through the site included NGOs, members of the Crisis Mappers Network,^13^ and health facility staff. Between January 29 and March 18, 2010, WHO/PAHO released 6 updated versions of the Master List. Each version of the MHFL included new health facilities, fewer duplicates, and corrected variable values (Table 2).

Haiti's master health facility list was posted to a public Google Site to increase the chances of it being used and updated over time.

The MHFL was used widely in the initial earthquake response. In addition to the Google Site, a link to the list was posted to many of the information portals that proliferated following the earthquake. The MHFL was also used as the reference data set for health facilities in the OpenStreetMap (OSM) platform.^14^^,^^15^ OSM updated its health facility layer with each of the 7 versions of the list.^16^

Online dissemination of the MHFL helped integrate the unique health facility codes into other data collection efforts.

As the initial effort transitioned from emergency response to reconstruction, stewardship of the MHFL was transferred to Shoreland, Inc., during April 2010.^17^ Following the cholera outbreak in Haiti in October 2010, fields for cholera treatment centers (CTCs) and cholera treatment units (CTUs) were added. The MSPP used the MHFL to determine which communities lacked health facilities so CTCs and CTUs could be installed to provide care to the affected population.^18^

In September 2011, the MSPP incorporated data from the MHFL into its routine health information system, which collects information on key services provided and human resources present at each facility. The MHFL formed the basis of the *Liste des Institutions Sanitaires*, the MSPP's listing of health facilities in the country,^19^ which is an updated and more robust version of the HSIS. It was also integrated into the *Carte Sanitaire*, the MSPP's service delivery and infrastructure status database.^20^^,^^21^ MEASURE Evaluation and the MSPP's UPE continue to work collaboratively to update and validate the *Liste des Institutions Sanitaires* in coordination with the directors of statistics and epidemiology within each of the 10 departments. Health facilities and field hospitals can now be uniquely identified. However, some duplicates and data quality issues remain.

Haiti's master health facility list developed for the earthquake response was eventually incorporated into the routine health information system.

## GOVERNANCE OF A NATIONAL MHFL: OPPORTUNITIES AND CHALLENGES

In routine health system planning, lists of health facilities, generally maintained by ministries of health, help organize information about health systems and are instrumental to answering basic questions such as how health services are distributed in a country and how resources may be allocated to address gaps in health service coverage.^22^^,^^23^ These lists facilitate reporting on the condition of health infrastructure and capacity to deliver services, which are key information requirements during a response to a humanitarian crisis, such as a natural disaster.^24^ Such lists are also essential for routine health information systems because they allow information about specific health facilities to flow within distributed networks in support of health decision-making.^25^^,^^26^

### Challenges

#### Multiple Sources of Information

In many countries, information about health facilities exists within stand-alone systems designed for discrete purposes. Lack of standardized naming conventions and codes unique to each facility but common across information systems introduces ambiguity to facility identity when comparing or consolidating multiple lists, resulting in duplications.^27^ It may be difficult to link multiple sources of information to support decision-making under normal circumstances, let alone during a disaster.^28^^,^^29^

#### Lack of Procedures

Ministries of health hold an essential ownership, management, verification, and communication function for MHFLs. Several dynamics explain why many ministries of health do not have an adequate MHFL. Procedures for regular updates may be lacking, causing information to easily become out-of-date. It is also common for facility-based health services in low- and middle-income countries to involve a complex array of multilateral, bilateral, public, and private for-profit and not-for-profit organizations.^30^ These organizations typically maintain information about the health facilities they support. However, there may be little or no information sharing among these groups or with the ministry of health. This is certainly the case in Haiti, where coordination between the government and the NGO community has historically been poor.^31^

Ministries of health play an essential role in creating, managing, and verifying data for master health facility lists.

#### Coordination

Haiti's MHFL provides a national-level view of Haiti's health facility infrastructure. The MHFL is updated at one central location by the UPE and consolidates information from the MSPP, WHO/PAHO, HHS/Centers for Disease Control and Prevention (CDC), the United States Agency for International Development (USAID), and the NGO community. Yet some coordination issues remain. Although publically available, there is a gap in regular updates to the MHFL and ongoing quality control efforts are necessary to maintain and improve the quality of the data and to remove duplicate records.

### Opportunities

#### Open Data and Social Networks

When MHFLs do not exist or are incomplete, responders during crises will need to collect data for immediate purposes. In the case of Haiti, open data, social networks, and volunteered geographic information were major factors that facilitated information flow about health facilities during the earthquake response.^15^^,^^32^^–^^37^ In addition, multiple organizations collected information directly from health facilities following the earthquake. However, lack of coordination among these organizations created confusion and overwhelmed health facility personnel. A pre-existing list that was updated at a central location could have mitigated this situation.

Open data, social networks, and volunteered geographic information are playing an increasing role in crisis response.

#### Quality Control Processes

The process of integrating data from multiple sources can spawn a proliferation of duplication and errors. For multi-sourced data to be widely accepted as reliable information, quality control processes must be in place to rapidly screen and verify data before it becomes official data. In the case of Haiti, central-level engagement of officers within the health system provided a quality check of the information in each of the iterations of the MHFL. However, validation can best occur with the engagement of appropriate staff at more local levels of health system administration.

#### Free, Online Access

Web-based repositories for MHFLs, such as Haiti's or Kenya's repositories, ensure that lists are available when needed and also can provide a platform for the maintenance of facility data over time.^3^^,^^21^^,^^38^ Ease of access to health facility lists increases the likelihood of data use. Data users and generators can then feed information to the system to create a cycle that should improve list completeness and quality over time.

The draft guidance from WHO on how to create an MHFL outlines a standardized process and provides WHO-endorsed standards for data format and data governance.^3^ The WHO guidelines also provide information on how the content of an MHFL can be made accessible and maintained over time.

## CONCLUSION

Having an accurate, regularly updated, and freely accessible national MHFL is important for effective routine planning and the delivery of health care services. During the 2010 Haiti earthquake response, the creation of a functioning MHFL proved useful for coordination and reconstruction efforts including subsequent events such as the cholera outbreak. A pre-populated data set that was comprehensive, accurate, and relatively up-to-date would have greatly facilitated initial relief efforts. Recognizing that disasters can occur anywhere and that accurate data are critical for effective response, countries without lists should develop and maintain an MHFL. Modest efforts in this area could greatly enhance the ability to mount a rapid, coordinated, and effective response.

## Notes

**Peer Reviewed**

**First published online:** 2014 Aug 5
